# Supplementary material for: Causal Relationship between Aging and Anorexia Nervosa: A White-Matter-Microstructure-Mediated Mendelian Randomization Analysis
Source: Biomedicines. 2024 Aug 16;12(8):1874. doi: 10.3390/biomedicines12081874 (PMC11351342; doi:10.3390/biomedicines12081874)
Supplement: Supplementary file 1 [file biomedicines-12-01874-s001.zip › Supplementary Figure/Figure S8/Leaveoneout plot of MR analysis for TL to ubm-b-1563.pdf]

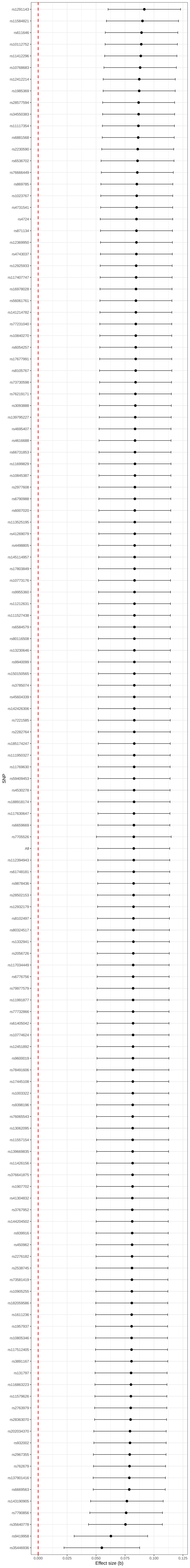
rs1291143
rs11584821
rs611646
rs10112752
rs11412296
rs10768683
rs12412214
rs1985369
rs28577594
rs34550383
rs11117354
rs6881568
rs2230590
rs6536702
rs76666449
rs869785
rs1023767
rs4731541
rs4724
rs871134
rs12369950
rs4743037
rs12925933
rs117407747
rs16978028
rs56061761
rs141214782
rs77231040
rs10840270
rs6054257
rs17677991
rs8105767
rs73730598
rs76219171
rs3093888
rs139795227
rs4695407
rs4616688
rs66731853
rs11699829
rs10845387
rs2977608
rs6790988
rs6007020
rs113525195
rs41269079
rs4498805
rs145114957
rs17803849
rs10773176
rs9955360
rs11212631
rs111527438
rs6584579
rs80116508
rs13230646
rs9940099
rs150150565
rs3785074
rs45604339
rs142426306
rs7221585
rs2282764
rs185174247
rs111950327
rs11769630
rs59409453
rs4530278
rs188918174
rs117630647
rs6659669
rs7705526
All
rs112394943
rs61748181
rs9878436
rs28502153
rs12932179
rs8102497
rs80324517
rs1332941
rs2056726
rs117034449
rs6776756
rs79977579
rs11991877
rs77732866
rs61405042
rs10774624
rs12451892
rs9600019
rs78491606
rs17445108
rs1003322
rs9398196
rs76065543
rs13062095
rs11557154
rs139669835
rs11426156
rs376641875
rs1907702
rs41304832
rs3767952
rs144204502
rs939916
rs450962
rs2276182
rs2538745
rs73581419
rs10905255
rs182059586
rs1611236
rs1957937
rs10805346
rs117512405
rs3891167
rs131797
rs116863223
rs11579626
rs2763979
rs28363070
rs202034370
rs932002
rs2967355
rs762679
rs137901416
rs6669563
rs143190905
rs7790856
rs35640778
rs9419958
rs35446936
SNP
0.000
0.025
0.050
0.075
0.100
0.125
Effect size (b)
